# Hygiene Measures and Decolonization of *Staphylococcus aureus* Made Simple for the Pediatric Practitioner

**DOI:** 10.1097/INF.0000000000004294

**Published:** 2024-02-26

**Authors:** Fabien Cane, Klara M. Posfay-Barbe, Laure F. Pittet

**Affiliations:** * From the Division of General Pediatrics, Department of Pediatric, Gynecology and Obstetrics, University Hospitals of Geneva and Faculty of Medicine, Geneva, Switzerland.

**Keywords:** *Staphylococcus aureus*, methicillin-resistant *Staphylococcus aureus*, methicillin-susceptible *Staphylococcus aureus*, decolonization, secondary prevention, pediatric

Methicillin-resistant *Staphylococcus aureus* (MRSA) and methicillin-susceptible *Staphylococcus aureus* are established as the main culprits for skin and soft tissue infections (SSTI).^1^ School-age children (particularly those 6 to 11 years old) have the highest incidence of *S. aureus* colonization and SSTI.^2^ *S. aureus* SSTI has a high propensity to recur, with recurrence rates ranging from 14% to 70%.^3–5^ Most recurrences (up to 90% in some series) are presumably caused by the same strain of *S. aureus*, with the same antimicrobial profile, emphasizing the important role individual and environmental colonization plays in reinfection.^6^

Colonization sites are usually the nose (in 100% of carriers), hands (90%), perineum (60%), pharynx (25%–50%) and skin (10%–25%).^7^ Most *S. aureus* strains involved in infection (over 80%) originate from the nasal mucosa,^8^ emphasizing the importance of nasal decolonization. *S. aureus* strains are also present in the environment, and are usually shared within the household; *S. aureus* colonization of the patient and/or their household members is a proven risk factor for *S. aureus* SSTI recurrences.^9^ Therefore, basic hygiene measures with or without decolonization regimen are key factors in preventing *S. aureus* SSTI recurrences. However, only a limited number of studies have investigated their efficacy.^10^ Hence, clinicians may feel uncertain about whether, when, how and for whom decolonization measures should be prescribed. The aim of this review is to summarize the evidence to inform clinical practice.

## What do “Hygiene Measures” Involve and Are They Evidence-based?

Any article addressing SSTI recurrence prevention mentions hygiene measures. These measures include avoiding contact with open wounds and contaminated surfaces, hand washing, frequent bathing, avoiding sharing personal hygiene items, keeping fingernails short and daily changing of pajamas, bed sheets, towels and washcloths.^11,12^ However, no study has ever assessed the effectiveness of those measures, their optimal frequency (eg, daily vs. weekly) or established which ones, if any, are genuinely beneficial.

## What Does the Literature on Pediatrics *S. aureus* Decolonization Show?

A recent systematic review summarizing studies assessing the efficacy of interventions to prevent recurrent staphylococcal SSTI in children retrieved only a few studies, with inconclusive findings.^13^ Five randomized control trials (RCTs) compared several decolonization strategies applied to patients with SSTI (index case), with or without their household members. The interventions included intranasal mupirocin, with or without chlorhexidine body washes or bleach baths. These interventions were compared with education on standard hygiene measures (control group), which was also provided to the intervention group.^13^

One RCT involving 987 children compared the prescription of bleach baths twice a week for 3 months to hygiene measures alone and failed to show a difference in SSTI recurrence [17.0%, 95% confidence interval: 13.8–20.6 vs. 20.9%, 95% confidence interval: 17.4–24.8].^14^ However, the trial excluded children with 4 or more recurrences of SSTI, and only 56% of the participants were colonized by *S. aureus* at inclusion.

**FIGURE 1.**
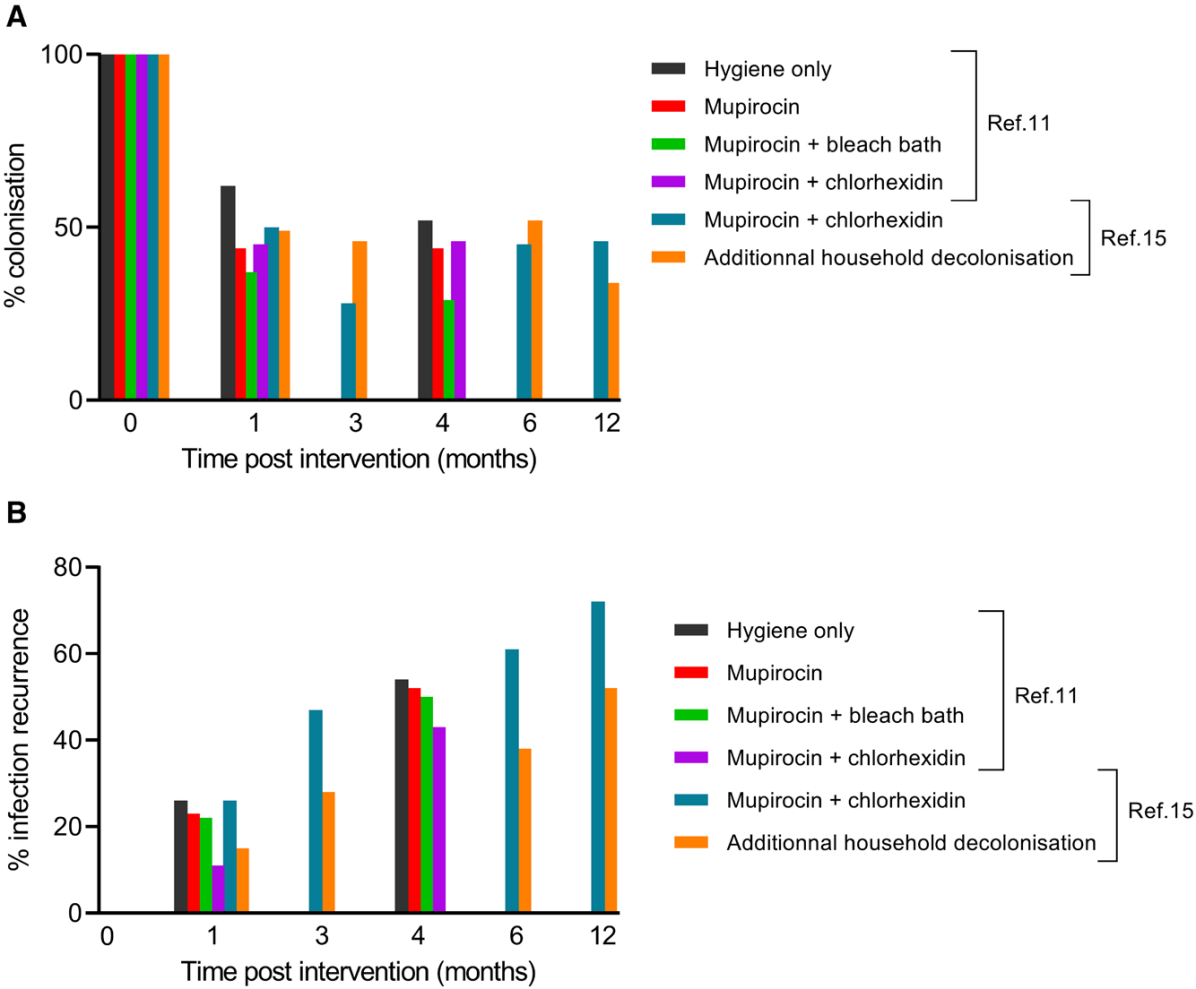
Prevalence of colonization and recurrence with different decolonization regimen.

A 4-group RCT compared the efficacy of 3 different 5-day decolonization protocols (intranasal mupirocin alone or combined with chlorhexidine washes, or with bleach baths) to hygiene measures alone for the clearance of *S. aureus* colonization and the prevention of SSTI recurrence in 300 children and adults.^11^ The decolonization rate was lower in the group allocated to hygiene measures alone (38% at the 1-month follow-up visit, vs. 55% to 63%) and was highest in the group allocated to intranasal mupirocin with bleach baths (71% at the 4-months follow-up visit; Fig. 1A). However, the rate of SSTI recurrence was similar across groups, with approximatively half of all participants experiencing a recurrence within the first 6 months of follow-up (Fig. 1B).

Another RCT involving 183 children with SSTI compared a 5-day decolonization protocol (intranasal mupirocin and chlorhexidine washes) prescribed either to the index case alone or to all household members.^15^ In the latter group, the authors observed a significant decrease in SSTI recurrence in the index case in the 3 to 12 months following decolonization (Fig. 1B), together with a reduction in SSTI occurrence in household members in the first 6 months of follow-up.

One 3-group RCT involving 223 children and adults compared hygiene measures alone to a 7-day household decolonization protocol (intranasal mupirocin and chlorhexidine washes), with or without daily reminders to increase compliance.^16^ The rate of recurrences was so low that the trial failed to find any difference between the groups. In secondary analyses, the authors found that self-reported full compliance with the decolonization protocol was associated with quicker clearance of colonization in the index case.

A 2-group RCT involving 102 children and their households compared a 5-day decolonization protocol (intranasal mupirocin and bleach baths) prescribed either to the whole household or restricted to household members who had an SSTI in the past year.^17^ The authors found no difference in the risk of SSTI recurrences. Trial limitations included an imbalance in the baseline MRSA colonization rate between the 2 groups and low compliance.

Hence, the systematic review concluded that decolonization measures appear to be somewhat helpful in clearing staphylococcal colonization, but are only marginally superior to hygienic measures alone, with an uncertain impact on SSTI recurrence. However, it is important to keep in mind that all 5 studies were performed in the USA, where *S. aureus* microbiology differs from Europe or Asia, particularly in terms of toxin production and antimicrobial sensitivity (eg, USA300).^13,18^ Decolonization rates are indeed lower in places with a higher prevalence of MRSA.^19,20^ Interestingly, a recent cohort study from the Netherlands found a significant association between failure to eradicate MRSA and ciprofloxacin resistance, although ciprofloxacin was not being used for decolonization.^21^

Whether systemic antibiotics should be used in addition to the decolonization protocol to prevent SSTI recurrences has not been well investigated and is usually not routinely recommended by guidelines. A few trials have investigated if systemic antibiotics could increase the decolonization rate in outpatient settings, without reporting on recurrences of infection.^22^ One RCT involving 98 adults with MRSA colonization evaluated a 7-day decolonization protocol (intranasal mupirocin and chlorhexidine washes) with or without oral rifampicin and clindamycin.^23^ Although the early decolonization rate was slighter higher in the group with systemic antibiotics, there was no difference at the 1-year follow-up. Another RCT involving 69 children and adults with MRSA throat colonization evaluated a 7-day decolonization protocol (intranasal mupirocin and chlorhexidine washes) with or without systemic antibiotics (oral rifampicin with either clindamycin or trimethoprim-sulfamethoxazole).^24^ At the 6-month follow-up the decolonization rate was substantially higher in patients who had received systemic antibiotics (61% vs. 13%).

## How to Prescribe Decolonization Measures?

In the absence of evidence, the frequency, duration and combination of the different decolonizing interventions varies between guidelines and hospitals.^13^ Decolonization protocols usually include a 5-day to 10-day regimen (but up to 3-month regimen) of daily chlorhexidine hair and body washes (with particular focus on areas *S. aureus* colonizes, ie, inguinal and axillary folds); and a 5- to 10-day regimen of twice to thrice daily intranasal mupirocin, applied with a single-use cotton swab into each nostril. Chlorhexidine can be substituted by octenidine, with the same efficacy profile and better tolerability.^25^ A 5- to 7-day regimen of twice daily chlorhexidine mouthwashes (or oral spray) can be added after teeth brushing to decolonize the oropharynx.^13^ In the presence of (recurrent) styes, a 7-day regimen of twice daily fusidic acid eye ointment can be tried. Bleach baths can be used as an alternative to chlorhexidine body wash in small children, as they are convenient and also more efficient to decolonize the inguinal folds in infants wearing diapers.^11^ Regular bathing in a chlorinated swimming pool is also effective in preventing SSTI and can be offered as an alternative.^26^ Decolonization protocols should always be prescribed together with the implementation of hygiene measures to limit the risk of recolonization. Moreover, as the treatment dries out the skin, the concomitant use of moisturizing emollients should be recommended to keep the skin barrier intact. To enhance compliance and comprehension of the decolonization protocol and hygiene measures, an illustrated information sheet can be provided to families (examples provided in various language, Supplemental Digital Content 1–15, http://links.lww.com/INF/F442; http://links.lww.com/INF/F443; http://links.lww.com/INF/F444; http://links.lww.com/INF/F445; http://links.lww.com/INF/F446; http://links.lww.com/INF/F447; http://links.lww.com/INF/F448; http://links.lww.com/INF/F449; http://links.lww.com/INF/F450; http://links.lww.com/INF/F451; http://links.lww.com/INF/F452; http://links.lww.com/INF/F453; http://links.lww.com/INF/F454; http://links.lww.com/INF/F455; http://links.lww.com/INF/F456).

## Pending Further Studies, When Are Decolonization and/or Hygiene Measures Indicated?

The benefit of *S. aureus* decolonization protocols has been well established in studies involving individuals (mainly adults) in preoperative settings,^27,28^ on dialysis,^29^ or critically ill.^22,30^ This review, however, focuses on community-acquired SSTI in healthy children. Despite the lack of supporting evidence, most guidelines recommend decolonization after the first recurrence of *S. aureus* SSTI (ie, a second episode).^13^ Some guidelines also recommend decolonization measures after 1 episode of SSTI if there is a history of SSTI in any other household member.^31^ A single infection, even if caused by a Panton-Valentine leucocidin-positive *S. aureus*, is not sufficient to recommend decolonization based on the current available data.^32^

## Should Decolonization Protocol be Used the Index Case or the Whole Household?

Children colonized with *S. aureus* are likely to live in an environment colonized by the same *S. aureus* strain.^9^ Interestingly, pet dogs and livestock also seem to be a reservoir for *S. aureus*, even though their role in the recurrence of SSTI is unclear.^33–37^

The usefulness of decolonizing a child who will likely be rapidly recolonized by their household member or environment is questionable. As discussed above, prescription of decolonization measures to all household members can reduce the risk of SSTI recurrence slightly, in both the index case and their household contacts.^15^ This, however, must be balanced with the tediousness of subjecting an entire household to highly burdensome measures, and therefore the risk of poor compliance with decolonization protocol and hygiene measures. Whether household decolonization should involve all members or be restricted to those with SSTI is still unclear.^17^ To the best of our knowledge, decolonization of pets has not been studied.

## Does the Acute Treatment of SSTI Influence the Risk of Recurrences?

The first step in preventing a recurrence of *S. aureus* infection is to successfully treat the acute infection. This entails incision and drainage if indicated (ie, for abscesses), and potentially addition of systemic antibiotics, particularly if the infection is severe (ie, signs of sepsis), extended (ie, multiple sites of infection), occurring in a high-risk patient (ie, immunocompromised patients), in an area difficult to drain (ie, genitalia, perianal region, etc.), associated with septic phlebitis or if incision has failed.^19^ The antibiotic regimen will be guided by local microbial ecology and infection severity, and can include beta-lactams, clindamycin, trimethoprim-sulfamethoxazole, linezolid, vancomycin or dalbavancin.^38^ The routine addition of rifampin is not recommended for SSTI.^39^

Whether the acute treatment of SSTI influences recurrences has been studied poorly. However, in 1 RCT involving 1013 patients above 12 years old with SSTI who had undergone drainage of an uncomplicated skin abscess, participants receiving trimethoprim-sulfamethoxazole had a lower risk of needing surgical drainage, new skin infections and infections in household members, compared with participants receiving an oral placebo.^40^ In an observational study of 383 children with SSTI, receiving systemic antibiotics after the drainage decreased the 1-year risk of SSTI recurrence; interestingly, clindamycin was more effective than trimethoprim-sulfamethoxazole in eradicating colonization and preventing SSTI recurrences.^41^

## How to Manage SSTI Recurrence After Decolonization?

When SSTI recurs despite a well-conducted decolonization protocol, the same decolonization regimen can be repeated or improved using the suggestions mentioned above.^13^ In particular, the decolonization protocol should involve the whole household if this has not been done before. The addition of systemic antibiotics should be considered as well, particularly in those with extranasal colonization.^22^ Stricter cleaning of objects that are shared within the home (eg, door handles or remote controls) could also be encouraged, although there is no data to support this.

## What Are the Arguments Against Decolonization?

In addition to their limited efficacy, decolonization measures are time-consuming and burdensome. Lack of compliance has been identified as a major limitation in most of the studies presented above, and was the reason why 1 study evaluated the efficacy of daily reminders to participants.^16^ Although motivation can be assumed to be proportionate to the disease burden in the household, health literacy is also a limiting factor. In the United States, a third of the population is considered to have limited health literacy, that is ability to understand and correctly apply health information.^42^ Another consideration is the financial cost (direct and indirect) of the decolonization measures, balanced against the cost of SSTI recurrences. Finally, the rise of mupirocin- and chlorhexidine-resistant *S. aureus* strains should be considered, although they appear to remain rare.^15^

## CONCLUSION

When treating a child with recurrent SSTI, it is crucial to provide hygiene education at the very least. Pending further studies, the prescription of decolonization measures can be tried in selected patients with recurrent SSTI and sufficiently motivated household members. The aim of the decolonization protocol is to interrupt the cycle of recurrent infections by reducing the burden of staphylococcal colonization.^13^ Parents’ compliance with decolonizing regimens is not guaranteed, but is key to success. The balance of burden-benefit should be assessed individually, and the family must be supported in their decolonization journey, for instance with a written protocol they can refer to at home. *S. aureus* decolonization protocols need to be studied further in children with recurrent SSTI, to identify the most effective strategies.

## ACKNOWLEDGMENTS

*The authors thank Pavle Manic for his help with the figures, and Gualtieri Renato, Heikkila Nelli, Lourenço Joao, Netea Stejara, Pavle Manic, Thøstesen Lisbeth, Turpitko Olexandr, Tzani Aimilia, Scherrer Sofia, Windisch Gabriella for the translation of information sheets for parents and patients.*

## Supplementary Material


