# Supplementary material for: Hygiene Measures and Decolonization of Staphylococcus aureus Made Simple for the Pediatric Practitioner
Source: Pediatr Infect Dis J. 2024 Feb 26;43(5):e178–82. doi: 10.1097/INF.0000000000004294 (PMC11003408; doi:10.1097/INF.0000000000004294)
Supplement: Supplementary file 1 [file inf-43-e178-s001.pdf]

STAPHYLOCOCCUS AUREUS
DECOLONIZATION PROTOCOL

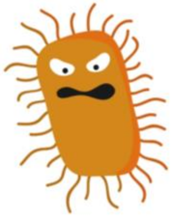

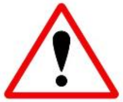

Wait for active lesions to heal before starting

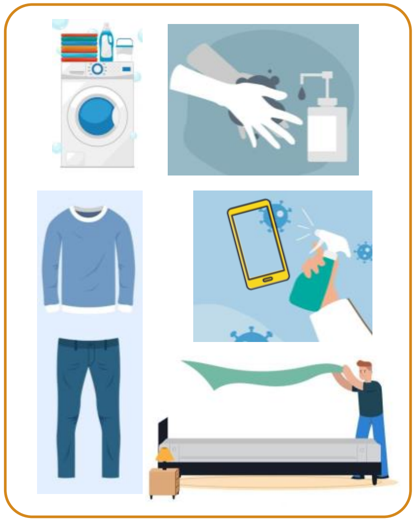

1/ Hygiene measures

- Short **fingernails**, clean **hands** with **liquid soap**
- **Clothes**, undies and pajamas **changed 1x/d**
- **Sheets** changed as often as possible, washed at **60°C**
- **Don't share** hygiene products (deodorant, brushes)
- **Shared objects disinfected** as often as possible

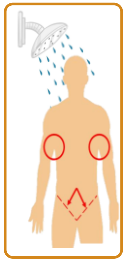

2/ Shower : Lifo Scrub ©

- **1x/d for 7 days**
- **Lather** and **leave on** for 2 minutes, insisting on creases (armpits and groin)
- Use **clean clothes** and sheets afterwards

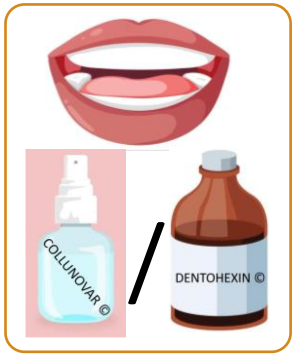

3/ Mouth : Dentohexine garg © or Collunovar spray ©

- **2x/d for 7 days**
- After **tooth brushing**,
    - **Gargle** month with oral solution
    - or use the **spray**
- **Dentures:** soak 30 minutes in disinfectant solution

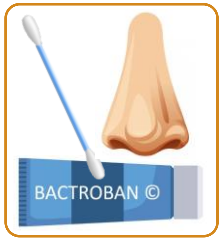

4/ Nose : Bactroban nasal ©

- **2x/d for 10 days**
- Using 1 clean **cotton swab** per side, massage a dab of ointment into the nasal cavity

5/ After decolonization

Continue to do the hygiene measures listed in point 1

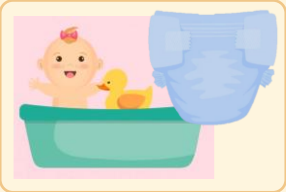

Chidren with **diapers**

- **Bleach baths** : 12ml/10L d'eau

Or

- **Swimming pool**

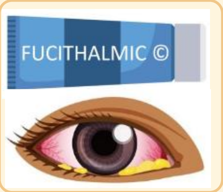

Recurrent **styes** : Fucithalmic gel©

- **2x/d for 7 days**
- Apply a small amount of gel to the **eyeball**
