# Supplementary material for: Hygiene Measures and Decolonization of Staphylococcus aureus Made Simple for the Pediatric Practitioner
Source: Pediatr Infect Dis J. 2024 Feb 26;43(5):e178–82. doi: 10.1097/INF.0000000000004294 (PMC11003408; doi:10.1097/INF.0000000000004294)
Supplement: Supplementary file 2 [file inf-43-e178-s002.pdf]

Hôpitaux Universitaires Genève

PROTOCOLE DE DECOLONISATION
DU STAPHYLOCOQUE DORE

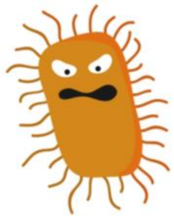

Ne pas commencer en cas d'infection active

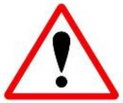

1/ Mesures d'hygiène

- Ongles **courts** et mains **propres** lavées au savon **liquide**
- **Vêtements**, sous-vêtements et pyjama **changés 1x/j**
- **Draps** changés le plus souvent possible, lavés à **60°C**
- Ne **pas partager** les produits d'hygiène (déo, brosses)
- **Objets communs désinfectés** le plus souvent possible

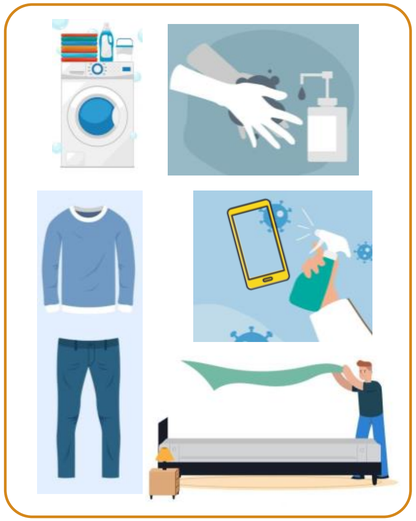

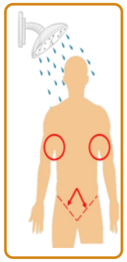

2/ Douche : Lifo Scrub ©

- **1x/j pendant 7 jours**
- Faire **mousser** et **laisser agir 2 minutes**, en insistant sur les **plis** (aisselles et aines)
- Utiliser vêtements et literie **propres** après

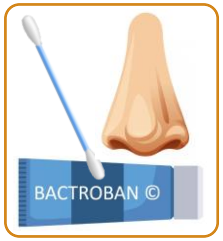

4/ Nez : Bactroban nasal ©

- **2x/j pendant 10 jours**
- Avec un **coton-tige propre** (1 par narine), appliquer une **noisette** de pommade dans la fosse nasale en **massant** la narine

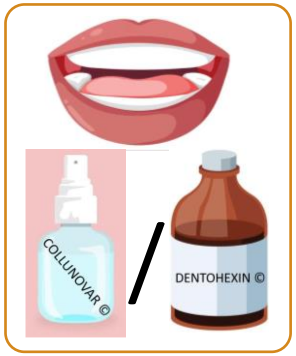

3/ Bouche : Dentohexine garg © ou Collunovar spray ©

- **2x/j pendant 7 jours**
- Après le **brossage de dents habituel**,
  - **gargariser** la bouche avec la solution orale
  - ou **asperger** avec le spray
- **Prothèses dentaires :** tremper 30 minutes dans une solution désinfectante

5/ Après la décolonisation

Continuer à appliquer les mesures d'hygiène listées au point 1

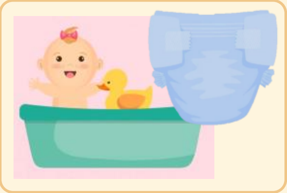

Enfants avec **couches**

- **Bains** avec **eau de Javel** : 12ml/10L d'eau

Ou

- **Piscine**

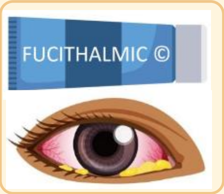

Orgelets à répétition : Fucithalmic gel ophtalmique ©

- **2x/j pendant 7 jours**
- Appliquer un peu de gel sur le **globe oculaire**
