# Supplementary material for: Hygiene Measures and Decolonization of Staphylococcus aureus Made Simple for the Pediatric Practitioner
Source: Pediatr Infect Dis J. 2024 Feb 26;43(5):e178–82. doi: 10.1097/INF.0000000000004294 (PMC11003408; doi:10.1097/INF.0000000000004294)
Supplement: Supplementary file 3 [file inf-43-e178-s003.pdf]

HUG Hôpitaux Universitaires Genève

# PROTOKOL FOR AFKOLONISERING
## AF STAPHYLOCOCCUS AUREUS

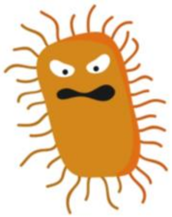

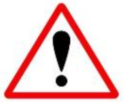

**Start ikke, hvis der er en aktiv infektion**

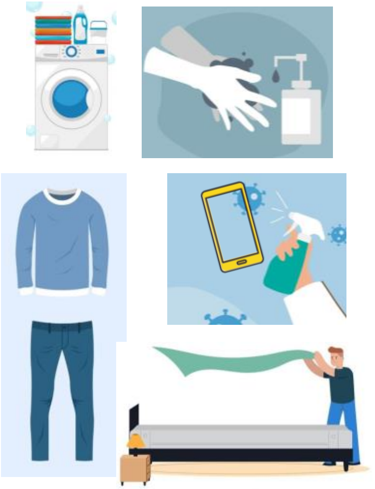

## 1/ Hygiejneforanstaltninger
- Korte negle og rene hænder vasket med flydende sæbe
- Tøj, undertøj og pyjamas skiftes dagligt
- Lagner skiftes så ofte som muligt, vaskes ved 60°C
- Hygiejneprodukter må ikke deles (deodoranter, børster)
- Fælles genstande desinficeres så ofte som muligt

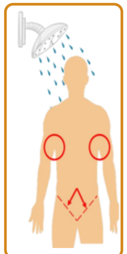

## 2/ Brusebad : Lifo Scrub ©
- **1 gang dagligt i 7 dage**
- Skum og lad virke i 2 minutter, koncentrer dig om folder (armhuler og lyske)
- Skift derefter til rent tøj og rent sengetøj

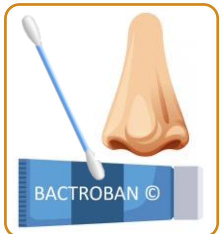


## 4/ Næse : Bactroban nasal ©
- **2 gange dagligt i 10 dage**
- Påfør med en ren vatpind en klat salve i næsehulen på hver side og massér næseboret

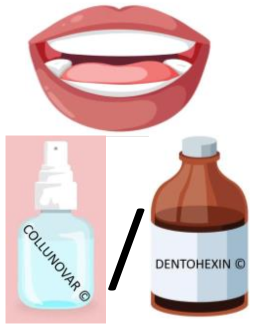


## 3/ Mund : Dentohexine garg © eller Collunovar spray ©
- **2 gange dagligt i 7 dage**
- Efter at have børstet tænder som normalt,
  - gurgle munden med den orale opløsning
  - eller spray i munden
- Protese: læg protesen i blød i 30 minutter i en desinficerende opløsning.

## 5/ Efter afkoloniseringen
Fortsæt med at anvende de hygiejneforanstaltninger, der er nævnt under punkt 1.

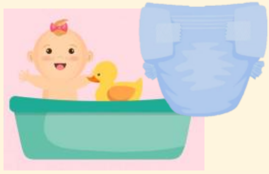

## Bleborn
- Bad i klorvand: 12 ml klor i 10 liter vand
- eller i svømmehallen

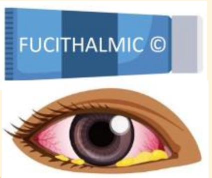


## **Gentagen øjeninfektion/bygkorn**: Fucithalmic øjengel ©
- 2 gange dagligt i 7 dage
- Påfør en smule gel på øjeæblet
