# Supplementary material for: Hygiene Measures and Decolonization of Staphylococcus aureus Made Simple for the Pediatric Practitioner
Source: Pediatr Infect Dis J. 2024 Feb 26;43(5):e178–82. doi: 10.1097/INF.0000000000004294 (PMC11003408; doi:10.1097/INF.0000000000004294)
Supplement: Supplementary file 4 [file inf-43-e178-s004.pdf]

PROTOCOL VOOR DEKOLONISATIE
VAN STAFYLOKOK AUREUS

Hôpitaux Universitaires Genève

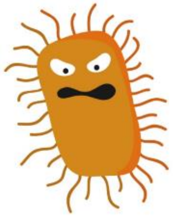

Niet starten als er een actieve infectie is

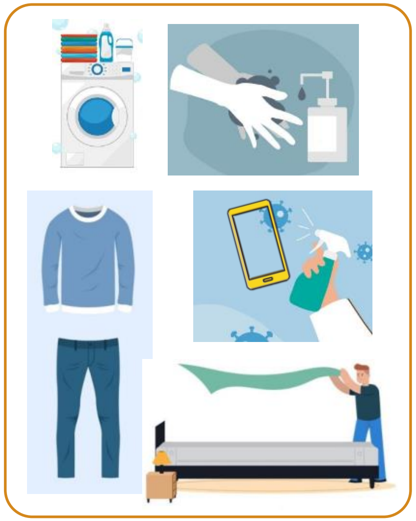

1/ Hygiënische maatregelen

- Korte nagels, handen wassen met vloeibare zeep
- Kleding, ondergoed en pyjama's 1x/d verschonen
- Lakens zo vaak mogelijk verschonen, wassen op 60°C
- Hygiëneproducten niet delen (deodorants, borstels)
- Gemeenschappelijke voorwerpen zo vaak mogelijk desinfecteren

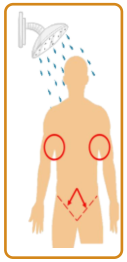

2/ Douche : Lifo Scrub ©

- **1x/dag gedurende 7 dagen**
- Laten schuimen en 2 minuten laten inwerken, vooral in de lichaamsplooien (oksels en liezen)
- Verschoon daarna kleding en beddengoed

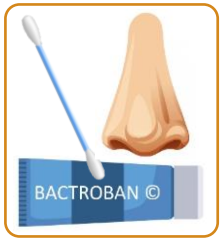

4/ Neus : Bactroban nasal ©

- **2x/d gedurende 10 dagen**
- Breng met één schoon wattenstaafje per kant een dotje zalf aan in de neusholte en masseer in het neusgat.

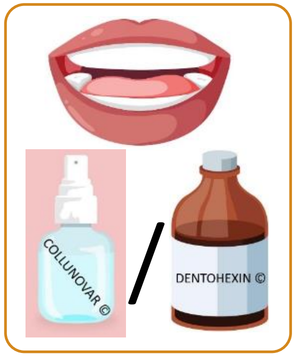

3/ Mond : Dentohexine garg © of Collunovar spray ©

- **2x/d gedurende 7 dagen**
- Na het tandenpoetsen,
  - gorgel de mond met de orale oplossing
  - of gebruik de spray
- Kunstgebit: 30 minuten laten weken in een desinfecterende oplossing

5/ Na de dekolonisatie

Blijf de hygiënemaatregelen van punt 1 toepassen

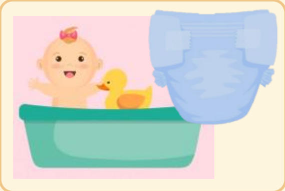

Kinderen met **luiers**

- Baden met bleekwater: 12ml/10L water

Of

- Zwembad

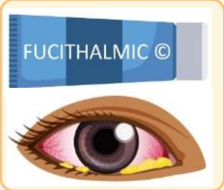

Herhaalde styes : Fucithalmic oftalmologische gel ©

- **2x/d gedurende 7 dagen**
- Breng een kleine hoeveelheid gel aan op de oogbol
