# Supplementary material for: Hygiene Measures and Decolonization of Staphylococcus aureus Made Simple for the Pediatric Practitioner
Source: Pediatr Infect Dis J. 2024 Feb 26;43(5):e178–82. doi: 10.1097/INF.0000000000004294 (PMC11003408; doi:10.1097/INF.0000000000004294)
Supplement: Supplementary file 5 [file inf-43-e178-s005.pdf]

Hôpitaux Universitaires Genève

STAPHYLOCOCCUS AUREUKSEN HÄÄTÖOHJEISTUS

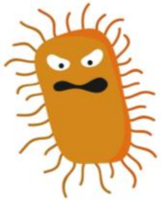

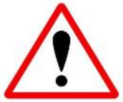

Älä aloita häätöhoitoa, jos sinulla on aktiivinen infektio tai tulehtunut haava

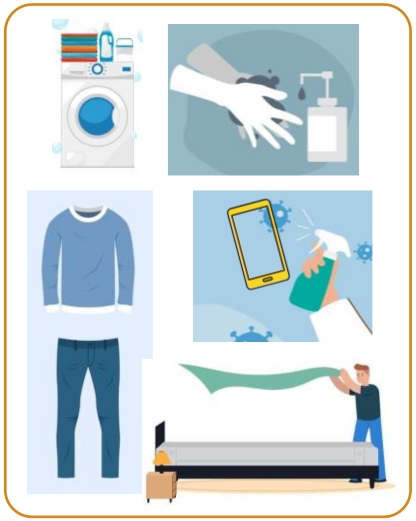

1/ Hygieniatoimenpiteet

- Lyhyet kynnet.
- Pese kädet nestesaippualla.
- Vaihda vaatteet, alusvaatteet ja pyjama 1 krt / vrk.
- Vaihda lakanat mahdollisimman usein, pese lakanat 60 °C:ssa.
- Älä jaa hygieniatuotteita (deodorantit, harjat) muiden kanssa.
- Desinfioi yhteisessä käytössä olevat esineet mahdollisimman usein.

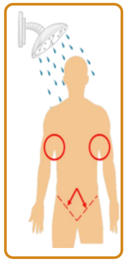

2/ Suihku : Lifo Scrub ©

- **1 krt /vrk, 7 päivän ajan**
- Vaahdota ja anna vaikuttaa 2 minuutin ajan keskittyen taipeisiin (kainalot ja nivuset).
- Vaihda puhtaat vaatteet ja vuodevaatteet pesun jälkeen.

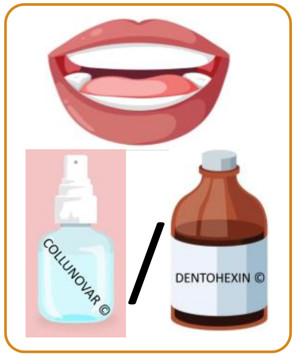

3/ Suu : Dentohexine garg © tai Collunovar spray ©

- **2 krt / vrk, 7 päivän ajan**
- Hammaspesun jälkeen:
  - kurlaa suu suuliuoksella
  - tai suihkuta suuhun
- Hammasproteesit:
  - liota 30 minuuttia desinfiointiliuoksessa.

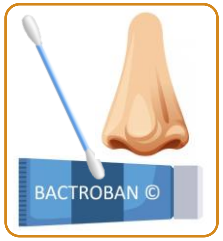

4/ Nenä : Bactroban nasal ©

- **2 krt / vrk, 10 päivän ajan**
- Levitä voidetta nenäonteloon puhtaalla pumpulipuikolla. Käytä erillistä pumpulipuikkoa kumpaankin sieraimeen.

5/ Häätöhoidon jälkeen

Jatka kohdassa 1 lueteltujen hygieniatoimenpiteiden noudattamista.

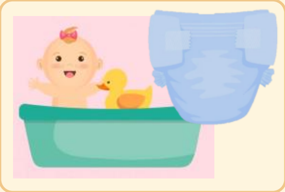

Lapset, joilla on vaipat

- Lisää valkaisuainetta kylpyveteen:
  - 12 ml / 10 l vettä.

Tai

- Käy säännöllisesti uima-altaassa.

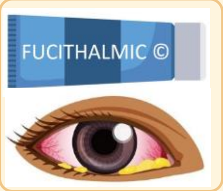

Toistuvat luomitulehdukset : Fucithalmic silmägeeli ©

- **2 krt / vrk, 7 päivän ajan**
- Levitä pieni määrä geeliä silmälle.
