# Supplementary material for: Hygiene Measures and Decolonization of Staphylococcus aureus Made Simple for the Pediatric Practitioner
Source: Pediatr Infect Dis J. 2024 Feb 26;43(5):e178–82. doi: 10.1097/INF.0000000000004294 (PMC11003408; doi:10.1097/INF.0000000000004294)
Supplement: Supplementary file 6 [file inf-43-e178-s006.pdf]

Hôpitaux Universitaires Genève

DEKOLONISATIONSPROTOKOLL VON STAPHYLOCOCCUS AUREUS

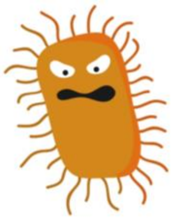

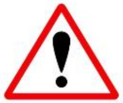

Nicht bei aktiver Infektion beginnen

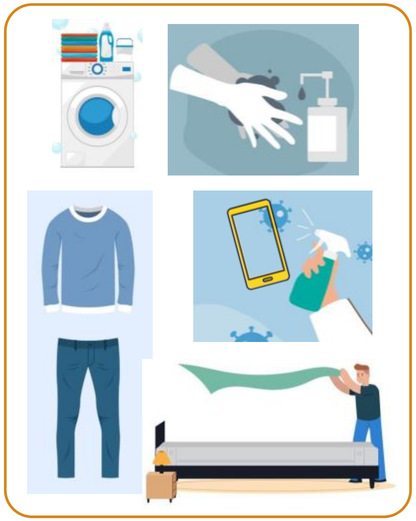

1/ Hygienemassnahmen

- **Kurze** Fingernägel und **saubere** Hände mit **Flüssigseife** gewaschen.
- Kleidung, Unterwäsche und Pyjama 1x/Tag **wechseln**
- **Bettwäsche** so oft wie möglich wechseln und bei **60°C** waschen.
- Hygieneprodukte **nicht teilen** (Deo, Bürsten)
- Gemeinsame Gegenstände so oft wie möglich **desinfiziert**

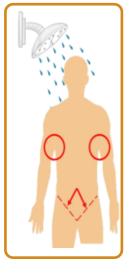

2/ Dusche : Lifo Scrub ©

- **1x/Tag während 7 Tagen**
- **Aufschäumen** und **2 Minuten einwirken** lassen, dabei besonders auf die **Falten** (Achseln und Leisten) achten.
- Hiernach **saubere** Kleidung und Bettwäsche anziehen.

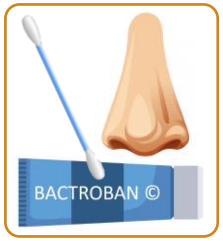

4/ Nez : Bactroban nasal ©

- **2x/Tag während 10 Tagen**
- Salbe (1cm) auf ein sauberes Wattestäbchen (1 pro Nasenloch) geben, in jedes Nasenloch applizieren und verreiben.

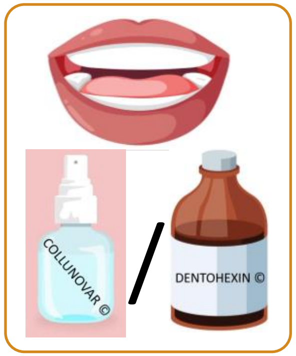

3/ Mund : Dentohexine garg © oder Collunovar spray ©

- **2x/Tag während 7 Tagen**
- Nach **dem üblichen Zähneputzen**,
  - den Mund mit der oralen Lösung **gurgeln**
  - oder mit dem **Spray besprühen**.
- **Zahnprothesen**: 30 Minuten in einer desinfizierenden Lösung einweichen

5/ nach der Dekolonisation

Die in Punkt 1 aufgelisteten Hygienemassnahmen weiterhin anwenden

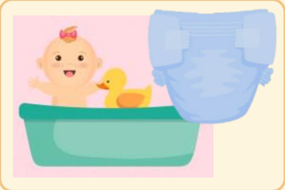

Kinder mit Windeln

- Bäder mit **Javelwasser**: 12ml/10L Wasser.

Oder

- **Schwimmbad**

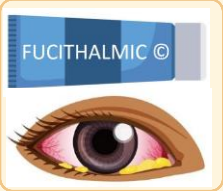

Wiederholte Gerstenkörner : Fucithalmic Augengel ©

- **2x/Tag während 7 Tagen**
- Etwas Gel in das zu behandelnde Auge geben
