# Supplementary material for: Hygiene Measures and Decolonization of Staphylococcus aureus Made Simple for the Pediatric Practitioner
Source: Pediatr Infect Dis J. 2024 Feb 26;43(5):e178–82. doi: 10.1097/INF.0000000000004294 (PMC11003408; doi:10.1097/INF.0000000000004294)
Supplement: Supplementary file 7 [file inf-43-e178-s007.pdf]

Hôpitaux
Universitaires
Genève

ΠΡΩΤΟΚΟΛΛΟ ΓΙΑ ΤΗΝ ΑΠΟΑΠΟΙΚΙΟΠΟΙΗΣΗ ΤΟΥ ΧΡΥΣΙΖΟΝΤΟΣ ΣΤΑΦΥΛΟΚΟΚΚΟΥ

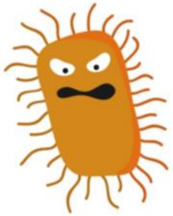

Δεν χρησιμοποιείται όσο υπάρχει ενεργή λοίμωξη

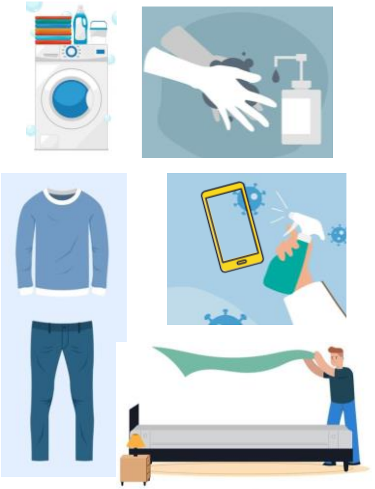

1/ Μέτρα υγιεινής

- Κοντά νύχια και καθαρά χέρια πλυμένα με υγρό σαπούνι
- Αλλαγή ρούχων, εσωρούχων και πιτζάμας 1x/ημέρα
- Αλλαγή σεντονιών όσο το δυνατόν συχνότερα, πλύσιμο στους 60°C
- Μην μοιράζεστε προϊόντα υγιεινής (αποσμητικά, βούρτσες)
- Απολύμανση κοινόχρηστων αντικειμένων όσο το δυνατόν συχνότερα

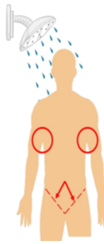

2/ **Ντους** : Lifo Scrub ©

- 1x/ημέρα για 7 ημέρες
- Κάντε το προϊόν να αφρίσει και αφήστε το για 2 λεπτά, επιμένοντας στις πτυχές (μασχάλες και βουβωνική χώρα).
- Χρησιμοποιήστε καθαρά ρούχα και τα κλινοσκεπάσματα αμέσως μετά.

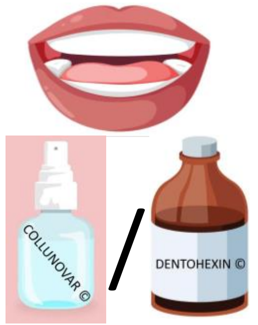

3/ **Στόμα** : Dentohexine garg © ή Collunovar spray ©

- **2x/ημέρα για 7 ημέρες**
- Αφού βουρτσίσετε τα δόντια σας ως συνήθως,
  - κάντε γαργάρες στο στόμα με το στοματικό διάλυμα
  - ή ψεκάστε στο στόμα
- Τεχνητές Οδοντοστοιχίες: μουλιάστε για 30 λεπτά σε απολυμαντικό διάλυμα

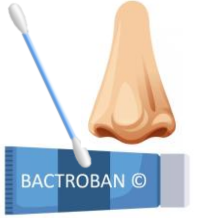

4/ **Μύτη** : Bactroban nasal ©

- **2x/ημέρα για 10 ημέρες**
- Χρησιμοποιώντας μια καθαρή μπατονέτα (μια για κάθε ρουθούνι), επαλείψτε στη ρινική κοιλότητα, ποσότητα αλοιφής όσο το μέγεθος ενός φουντουκιού, κάνοντας στη συνέχεια μασάζ στο ρουθούνι.

5/ Μετά την αποαποικιοποίηση

Συνεχίστε να εφαρμόζετε τα μέτρα υγιεινής που αναφέρονται στο σημείο 1.

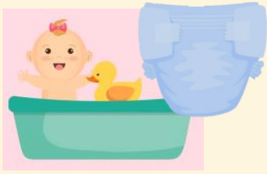

Παιδιά με **πάνες**

- Λουτρά με χλωρίνη: 12ml/10L νερού

Ή

- Πισίνα

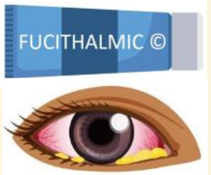

Επαναλαμβανόμενα κριθαράκια: Fucithalmic gel ophtalmique ©

- **2x/ημέρα για 7 ημέρες**
- Εφαρμόστε μικρή ποσότητα τζελ στο βολβό του ματιού
