# Supplementary material for: Hygiene Measures and Decolonization of Staphylococcus aureus Made Simple for the Pediatric Practitioner
Source: Pediatr Infect Dis J. 2024 Feb 26;43(5):e178–82. doi: 10.1097/INF.0000000000004294 (PMC11003408; doi:10.1097/INF.0000000000004294)
Supplement: Supplementary file 8 [file inf-43-e178-s008.pdf]

Hôpitaux Universitaires Genève

A DEKOLONIZÁCIÓS PROTOKOLL

STAPHYLOCOCCUS AUREUS

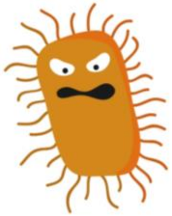

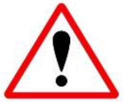

Ne kezdje el, ha aktív fertőzés van

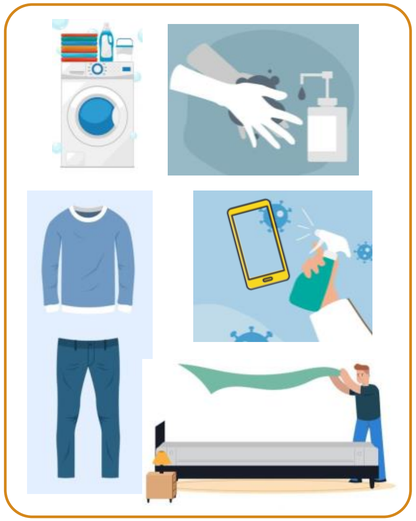

1/ Higiéniai intézkedések

- Rövid és tiszta körmök, folyékony szappannal mosott kéz
- Ruházat, alsónemű és pizsama cseréje 1x/naponta
- Lepedőcsere a lehető leggyakrabban, 60°C-on mosva
- Ne osszák meg egymással a higiéniai termékeket (dezodorok, kefék, stb)
- A közös tárgyakat a lehető leggyakrabban fertőtlenítsék

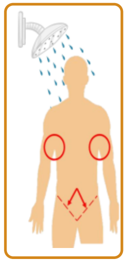

2/ Zuhany : Lifo Scrub ©

- **1x/nap, 7 napig**
- Habosítsa fel és hagyja rajta 2 percig, a hónalj és ágyékra koncentrálva
- Utána tisztítsa meg a ruhákat és az ágyneműt

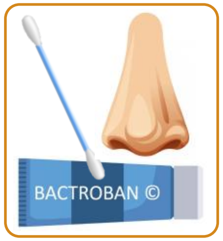

4/ Orr : Bactroban nasal ©

- **2x/nap, 10 napig**
- Oldalanként egy-egy tiszta vattapamacsot használva kenőcsöt kenjen az orrüregbe, masszírozva az orrlyukat

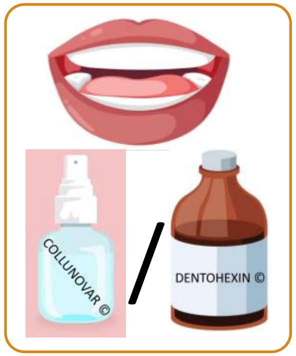

3/ Száj : Dentohexine garg © vagy Collunovar spray ©

- **2x/nap, 7 napig**
- A szokásos fogmosás után,
    - gargalizáljon a szájban a szájoldattal
    - vagy permetezze be a száját
- Fogsor: 30 percig áztassa fertőtlenítő oldatban

5/ A dekolonizáció után

Folytassa az 1. pontban felsorolt higiéniai intézkedések alkalmazását

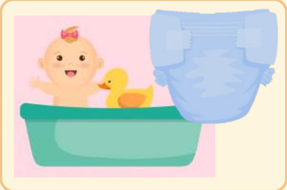

Pelenkás gyermekek

- Fürdő fehérítővel: 12ml/10L vízzel

Vagy

- Úszómedence

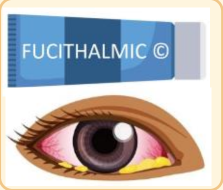

Ismételt szemfertözés:

Fucithalmic szemészeti kenyöcs ©

- **2x/nap, 7 napig**
- Rakjon egy kis kenőcsöt a szemgolyóra
