# Supplementary material for: Hygiene Measures and Decolonization of Staphylococcus aureus Made Simple for the Pediatric Practitioner
Source: Pediatr Infect Dis J. 2024 Feb 26;43(5):e178–82. doi: 10.1097/INF.0000000000004294 (PMC11003408; doi:10.1097/INF.0000000000004294)
Supplement: Supplementary file 9 [file inf-43-e178-s009.pdf]

# PROTOCOLLO PER LA DECOLONIZZAZIONE DELLO STAFILOCOCCO AUREO

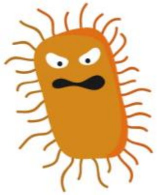

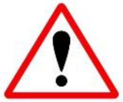

**Non iniziare in caso di un'infezione attiva**

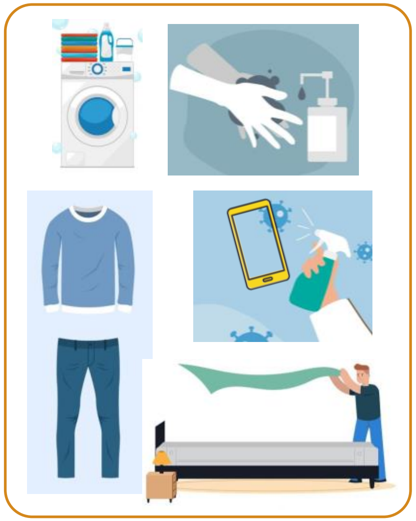

## 1/ Misure igieniche

- Unghie **corte** e mani **pulite** lavate con sapone **liquido**
- **Vestiti**, biancheria intima e pigiami **cambiati 1x/die**
- **Lenzuola** cambiate il più spesso possibile, **lavate a 60°C**
- **Non condividere** i prodotti per l'igiene (deodoranti, spazzole)
- **Disinfettare gli oggetti comuni** il più spesso possibile

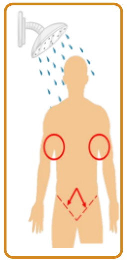

## 2/ Doccia : Lifo Scrub ©

- **1x/die per 7 giorni**
- Applicare la **schiuma** e lasciare **agire per 2 minuti**, concentrandosi sulle **pieghe cutanee** (ascelle e inguine).
- Dopo, utilizzare vestiti e biancheria da letto **puliti**

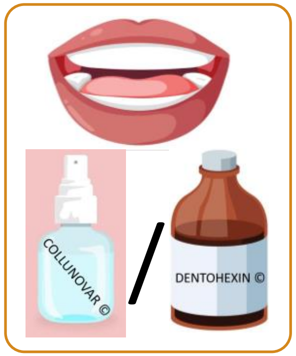


## 3/ Bocca : Dentohexine garg © o Collunovar spray ©

- **2x/die per 7 giorni**
- Dopo aver lavato i denti **come di consueto**,
  - fare dei **gargarismi** con la soluzione orale
  - o **spruzzare** in bocca
- **Protesi dentarie**: immergere per 30 minuti in una soluzione disinfettante

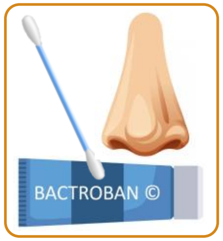


## 4/ Naso : Bactroban nasal ©

- **2x/die per 10 giorni**
- Con un cotton fioc pulito per lato, applicare una piccola quantità di pomata nella cavità nasale, massaggiando la narice.

## 5/ Dopo la decolonizzazione

Continuare ad applicare le misure igieniche elencate al punto 1.

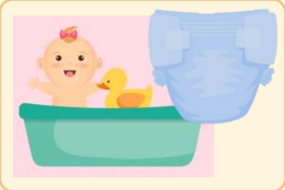

## Bambini con **pannolini**

- **Bagni con candeggina**: 12ml/10L di acqua

O

- **Piscina**

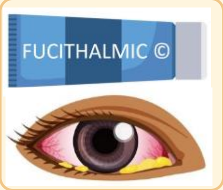


## Orzaiolo ricorrente : Fucithalmic gel oftalmico ©

- **2x/die per 7 giorni**
- Applicare una piccola quantità di gel sul **bulbo oculare**
