# Supplementary material for: Hygiene Measures and Decolonization of Staphylococcus aureus Made Simple for the Pediatric Practitioner
Source: Pediatr Infect Dis J. 2024 Feb 26;43(5):e178–82. doi: 10.1097/INF.0000000000004294 (PMC11003408; doi:10.1097/INF.0000000000004294)
Supplement: Supplementary file 10 [file inf-43-e178-s010.pdf]

HUG Hôpitaux Universitaires Genève

PROTOCOLO DE DESCOLONIZAÇÃO
DE STAPHYLOCOCCUS AUREUS

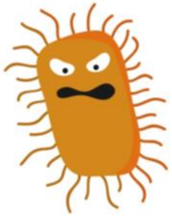

Não iniciar se houver uma infeção ativa

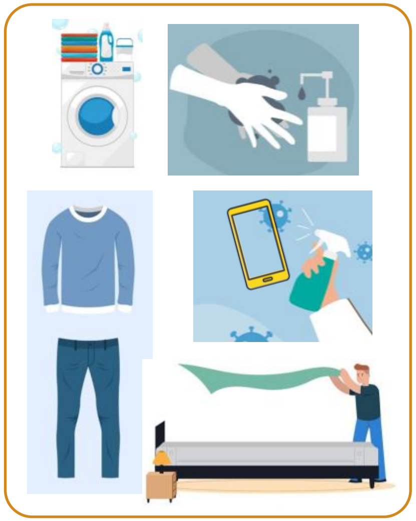

1/ Medidas de higiene

- Unhas **curtas** e mãos **limpas**, lavadas com sabão **líquido**
- **Mudança** de roupa, roupa interior e pijama **1x/d**
- **Mudar** os lençóis com a maior frequência possível, lavados a **60°C**
- **Não partilhar** os produtos de higiene (desodorizantes, escovas)
- Os objectos comuns são **desinfectados** com a maior frequência possível

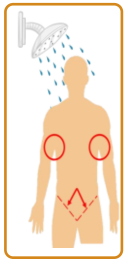

2/ Duche : Lifo Scrub ©

- **1x/dia durante 7 dias**
- Ensaboar e deixar atuar durante **2 minutos**, concentrando-se nas **dobras** (axilas e virilhas)
- **Limpar** depois a roupa e a roupa de cama

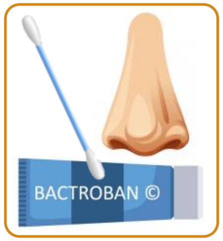

4/ Nariz : Bactroban nasal ©

- **2x/d durante 10 dias**
- Com um **cotonete limpo** de cada lado, aplicar uma gota de pomada na cavidade nasal, massajando a narina.

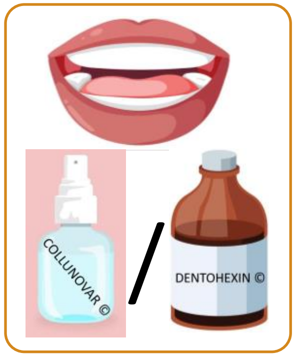

3/ Boca : Dentohexine garg © ou Collunovar spray ©

- **2x/d durante 7 dias**
- Depois de escovar os dentes,
    - **gargarejar** a boca com a solução oral
    - ou **pulverizar** na boca
- **Dentaduras**: deixar de molho durante 30 minutos numa solução desinfetante

5/ Após a descolonização

Continuar a aplicar as medidas de higiene enumeradas no ponto 1

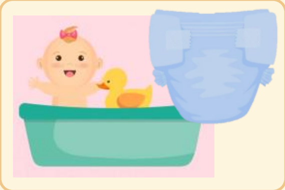

Crianças com **fraldas**

- **Banhos com lixívia**: 12ml/10L de água

Ou

- **Piscina**

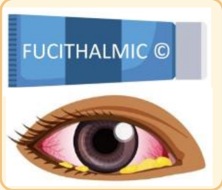

Hordéolo (terçol) com repetição : Fucithalmic gel oftálmico ©

- **2x/d durante 7 dias**
- Aplicar um pouco de gel no globo ocular
