# Supplementary material for: Hygiene Measures and Decolonization of Staphylococcus aureus Made Simple for the Pediatric Practitioner
Source: Pediatr Infect Dis J. 2024 Feb 26;43(5):e178–82. doi: 10.1097/INF.0000000000004294 (PMC11003408; doi:10.1097/INF.0000000000004294)
Supplement: Supplementary file 11 [file inf-43-e178-s011.pdf]

Hôpitaux Universitaires Genève

PROTOCOL DE DECOLONIZARE A STAFILOCOCULUI AURIU

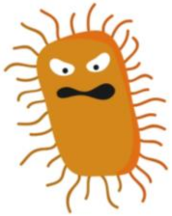

Nu începeți dacă există o infecție activă

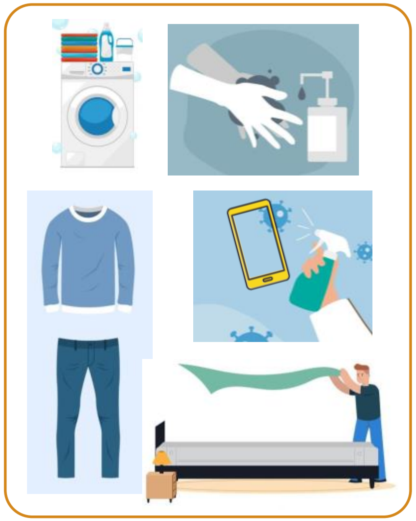

1/ Măsuri de igienă

- Unghii scurte și mâini curate și spălate cu săpun lichid
- Haine, lenjerie de corp și pijamale schimbate o dată pe zi
- Lenjerie schimbată cât mai des posibil, spălată la 60°C
- Nu împărțiți produsele de igienă (deodorante, perii)
- Obiecte comune dezinfectate cât mai des posibil

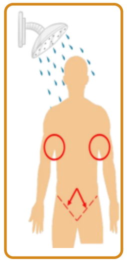

2/ Duș : Lifo Scrub ©

- **1x/zi timp de 7 zile**
- Faceți spumă și lăsați să acționeze timp de 2 minute, concentrându-vă pe pliuri (axile și inghinali)
- Curățați hainele și lenjeria de pat după aceea

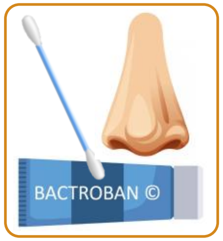

4/ Nas : Bactroban nasal ©

- **2x/zi timp de 10 zile**
- Folosind câte un tampon de vată curat pe fiecare parte, aplicați o picătură de unguent în cavitatea nazală, masând nara.

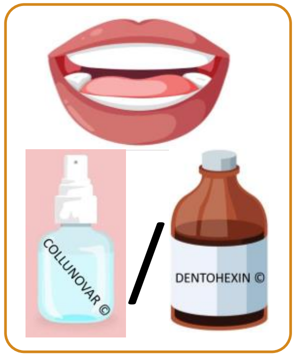

3/ Igiena orala : Dentohexine garg © sau Collunovar spray ©

- **2x/zi timp de 7 zile**
- După ce vă spălați pe dinți ca de obicei,
  - faceți gargară în gură cu soluția orală
  - sau pulverizați pe gură
- Proteze dentare: înmuiați timp de 30 de minute într-o soluție dezinfectantă

5/ După decolonizare

Continuați să aplicați măsurile de igienă enumerate la punctul 1.

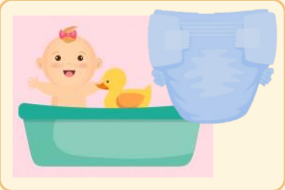

Copii cu **scutece**

- Băi cu înălbitor: 12ml/10L de apă

Sau

- Piscină

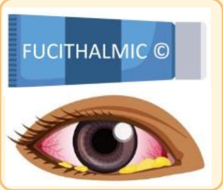

Ulcior (orjelete) repetat: gel oftalmic (e.g. Fucithalmic©)

- **2x/zi timp de 7 zile**
- Aplicați puțin gel pe globul ocular
