# Supplementary material for: Hygiene Measures and Decolonization of Staphylococcus aureus Made Simple for the Pediatric Practitioner
Source: Pediatr Infect Dis J. 2024 Feb 26;43(5):e178–82. doi: 10.1097/INF.0000000000004294 (PMC11003408; doi:10.1097/INF.0000000000004294)
Supplement: Supplementary file 12 [file inf-43-e178-s012.pdf]

HUG Hôpitaux Universitaires Genève

ПРОТОКОЛ ДЕКОЛОНИЗАЦИИ
ЗОЛОТИСТОГО СТАФИЛОКОККА

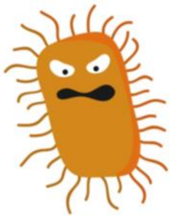

Не начинайте, если есть активная инфекция

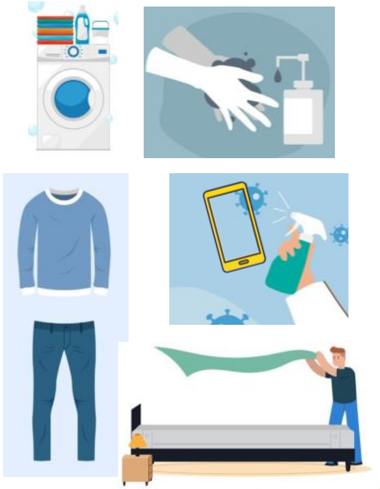

1/ Гигиенические меры
- Короткие ногти и чистые руки, вымытые с жидким мылом
- Одежда, нижнее белье и пижама меняются 1 раз в день
- Простыни менять как можно чаще, стирать при 60°C
- Не пользуйтесь общими средствами гигиены (дезодорант, зубные щетки)
- Общие предметы дезинфицируйте как можно чаще

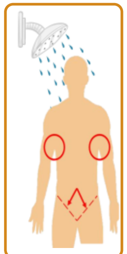

2/ **Душ** : Lifo Scrub ©
- **1 раз в день в течение 7 дней**
- Вспеньте и оставьте на 2 минуты, уделяя особое внимание складкам (в подмышках и в паху).
- После этого сменить чистую одежду и постельное белье

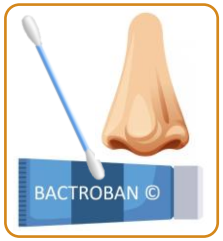

4/ **Нос** : Bactroban nasal ©
- **2 раза в день в течение 10 дней**
- С помощью чистой ватной палочки нанесите небольшое количество мази в полость носа, массируя ноздрю.

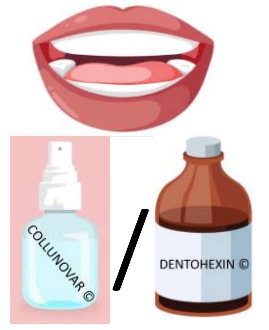

3/ **Рот** : Dentohexine garg © или Collunovar spray ©
- **2 раза в день в течение 7 дней**
- После чистки зубов, как обычно,
  - полоскать рот раствором для полости рта
  - или обработать спреем
- Зубные протезы: замочите на 30 минут в дезинфицирующем растворе

5/ После деколонизации
Продолжайте применять гигиенические меры, перечисленные в пункте 1

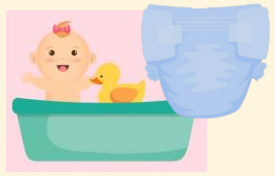

Дети с подгузниками
- Ванны с отбеливателем: 12 мл/10 л воды

Или
- Бассейн

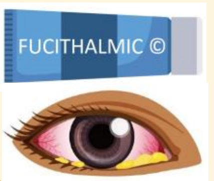

Повторные ячмени: Fucithalmic ophtalmic gel ©
- **2 раза в день в течение 7 дней**
- Нанесите немного геля на глазное яблоко
