# Supplementary material for: Hygiene Measures and Decolonization of Staphylococcus aureus Made Simple for the Pediatric Practitioner
Source: Pediatr Infect Dis J. 2024 Feb 26;43(5):e178–82. doi: 10.1097/INF.0000000000004294 (PMC11003408; doi:10.1097/INF.0000000000004294)
Supplement: Supplementary file 13 [file inf-43-e178-s013.pdf]

PROTOKOL O DEKOLONIZACIJI
ZLATNOG STAFILOKOKA

Hôpitaux
Universitaires
Genève

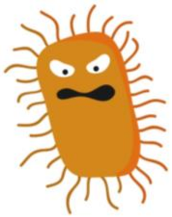

Ne započeti u slučaju aktivne infekcije

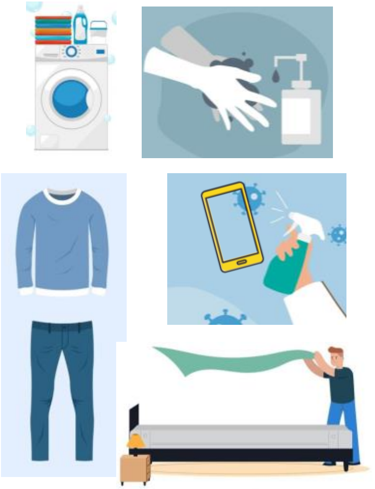

1/ Higijenske mere

- Kratki nokti i čiste ruke oprane tečnim sapunom
- Odeću, donji veš i pidžamu menjati jednom dnevno
- Čaršave menjati što češće, i prati na 60°C
- Higijenske proizvode ne deliti (dezodorans, četke)
- Uobičajene predmete dezinfikovati što češće

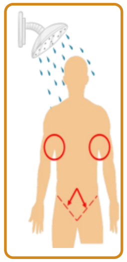

2/ Tuš : Lifo Scrub ©

- **Jednom dnevno tokom 7 dana**
- Zapeniti i ostaviti da deluje 2 minuta, fokusirajući se na pregibe (pazuhe i prepone)
- Čista odeća i posteljina nakon toga

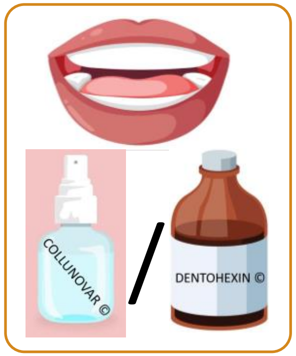

3/ Usta : Dentohexine garg © ili Collunovar spray ©

- **2 puta dnevno tokom 7 dana**
- Nakon pranja zuba,
    - oralnim rastvorom isprati usta
    - ili prskati sprejom
- Zubne proteze: potopiti 30 minuta u rastvoru za dezinfekciju

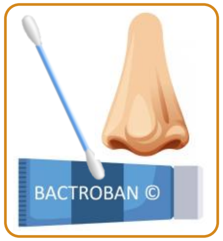

4/ Nos : Bactroban nasal ©

- **2 puta dnevno tokom 10 dana**
- Čistim štapićem za uši za svaku stranu naneti malu količinu pomade u nozdrvu, masirajući je

5/ Posle dekolonizacije

Nastaviti primenjivanje higijenskih mera navedene pod tačkom broj 1

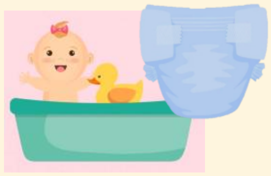

Deca sa pelenama

- Kupanje sa izbeljivačem: 12ml/10L vode

Ili

- Bazen

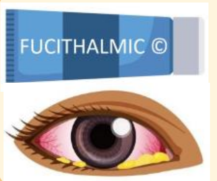

Hordeolum : Fucithalmic oftalmički gel ©

- 2 puta dnevno tokom 7 dana
- Naneti malo gela na očnu jabučicu
