# Supplementary material for: Hygiene Measures and Decolonization of Staphylococcus aureus Made Simple for the Pediatric Practitioner
Source: Pediatr Infect Dis J. 2024 Feb 26;43(5):e178–82. doi: 10.1097/INF.0000000000004294 (PMC11003408; doi:10.1097/INF.0000000000004294)
Supplement: Supplementary file 14 [file inf-43-e178-s014.pdf]

PROTOCOLO DE DESCOLONIZACIÓN DE STAPHYLOCOCCUS AUREUS

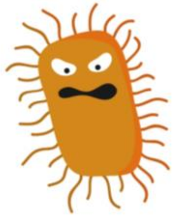

Hôpitaux Universitaires Genève

No empezar si hay una infección activa

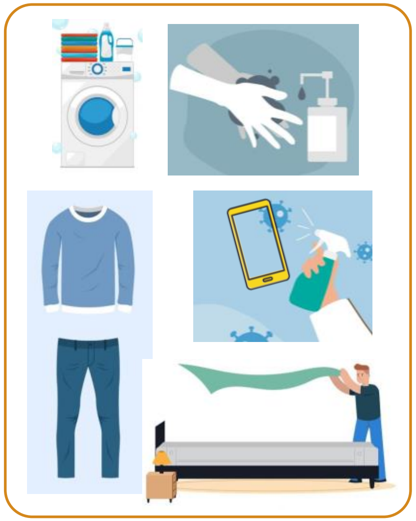

1/ Medidas de higiene

- Uñas **cortas** y manos **limpias** lavadas con jabón **líquido**
- **Cambio** de ropa, ropa interior y pijamas 1 vez al día
- **Sábanas** cambiadas lo más a menudo posible, lavadas a **60°C**
- **No compartir** productos de higiene (desodorantes, cepillos)
- **Objetos comunes desinfectados** lo más a menudo posible

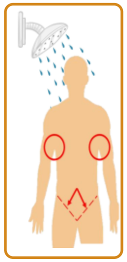

2/ Ducha : Lifo Scrub ©

- **1 vez al día durante 7 días**
- Hacer **espuma** y dejar **actuar durante 2 minutos**, insistiendo en los **pliegues** (axilas e ingles)
- Despues, uzar ropa y ropa de cama limpia

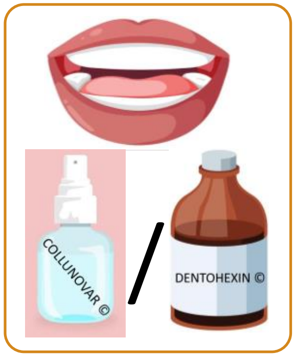

3/ Boca : Dentohexine garg © o Collunovar spray ©

- **2 veces al día durante 7 días**
- Después de cepillarse los dientes, hacer
  - **gárgaras** en la boca con la solución oral
  - o **pulverizar** en la boca
- **Prótesis dentales**: dejar en remojo durante 30 minutos en una solución desinfectante

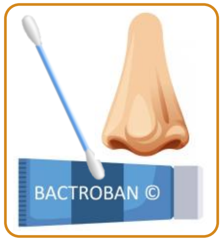

4/ Nariz : Bactroban nasal ©

- **2 veces al día durante 10 días**
- En cada fosa nasal, aplique un poco de pomada, con un bastoncillo de algodón limpio y masajee de cada lado

5/ Tras la descolonización

Seguir aplicando las medidas de higiene enumeradas en el punto 1

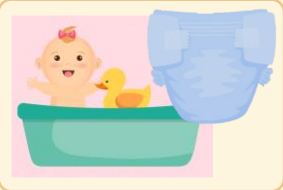

Niños con pañales

- **Baños de lejía**: 12ml/10L de agua

O

- **Piscina**

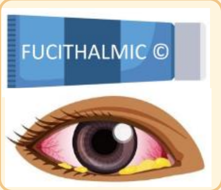

Orzuelos repetidos : Fucithalmic gel oftálmico ©

- **2 veces al dia durante 7 días**
- Aplicar un poco de gel en el **globo ocular**
