# Supplementary material for: Hygiene Measures and Decolonization of Staphylococcus aureus Made Simple for the Pediatric Practitioner
Source: Pediatr Infect Dis J. 2024 Feb 26;43(5):e178–82. doi: 10.1097/INF.0000000000004294 (PMC11003408; doi:10.1097/INF.0000000000004294)
Supplement: Supplementary file 15 [file inf-43-e178-s015.pdf]

Hôpitaux Universitaires Genève

ПРОТОКОЛ ДЕКОЛОНІЗАЦІЇ ЗОЛОТИСТОГО СТАФІЛОКОКУ

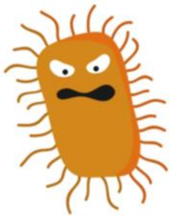

Не починати, якщо є активна інфекція

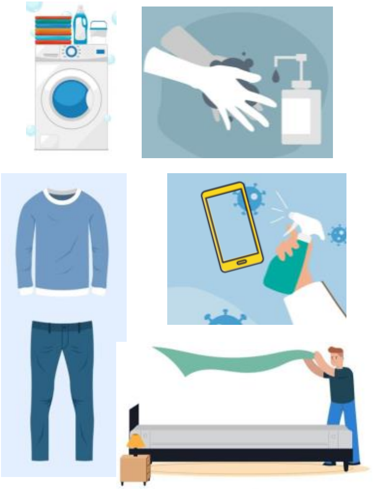

1/ Гігієнічні заходи

- Мати завжди коротко обрізані нігті та чисто вимиті з рідким милом руки
- Одяг, нижня білизна та піжами змінювати 1 раз на день
- Постільну білизну змінювати якомога частіш та прати при температурі 60°C
- Не користуватися спільними засобами гігієни (дезодорант, щітки для зубів)
- Предмети спільного користування дезінфікувати якомога частіше

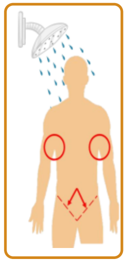

2/ Душ : Lifo Scrub ©

- **Приймати 1 раз на день протягом 7 днів**
- Спінити і залишити на 2 хвилини, концентруючись на складках (пахви і пах)
- Після цього випрати одяг та постільну білизну

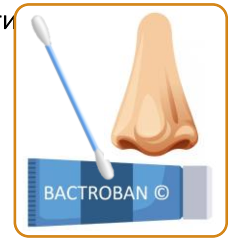

4/ Ніс : Bactroban nasal ©

- **2 рази на день протягом 10 днів**
- Наносити мазь чистою ватною паличкою у носову порожнину з обох боків , масажуючи ніздрю.

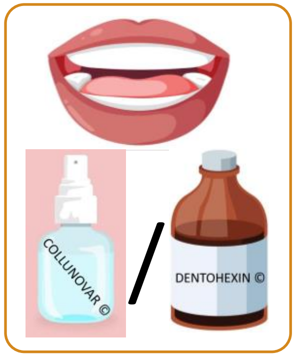

3/ Рот : Dentohexine garg © або Collunovar spray ©

- **2 рази на день протягом 7 днів**
- Після звичайного чищення зубів,
  - прополоскати ротову порожнину розчином для перорального застосування
  - або спреєм
- Зубні протези: замочити на 30 хвилин у дезінфікуючому розчині

5/ Після деколонізації

Продовжувати застосовувати гігієнічні заходи, перелічені в пункті 1

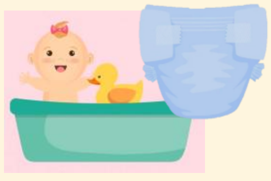

Діти з підгузками

- Ванни з відбілювачем: 12 мл/10 л води

Або

- Басейн

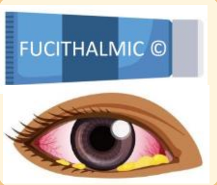

Повторювані ячміні : Fucithalmic ophtalmic gel ©

- **2 рази на день протягом 7 днів**
- Наносити трохи гелю на очне яблуко
